# Impact of Reinforcement Ratio on Shear Behavior of I-Shaped UHPC Beams with and without Fiber Shear Reinforcement

**DOI:** 10.3390/ma13071525

**Published:** 2020-03-26

**Authors:** Altug Yavas, Cumali Ogun Goker

**Affiliations:** Department of Civil Engineering, Balikesir University, 10145 Balikesir, Turkey; ogungoker@gmail.com

**Keywords:** steel fiber ultra-high performance concrete, reinforced concrete beam, shear behavior, end-hooked steel fiber

## Abstract

In the presented paper, the impacts of steel fiber use and tensile reinforcement ratio on shear behavior of Ultra-High Performance Concrete (UHPC) beams were investigated from the point of different tensile reinforcement ratios. In the scope of the experimental program, a total of eight beams consisting of four reinforcement ratios representing low to high ratios ranged from 0.8% to 2.2% were casted without shear reinforcement and subjected to the four-point loading test. While half of the test beams included 30 mm end-hooked steel fibers (SF-UHPC) with 2.0 vol%, the remaining beams were produced without the fiber to show possible effectiveness of the fiber use. The shear performances were discussed in terms of the load—deflection response, cracking pattern and failure mode, first cracking load and ultimate shear strength. In this sense, all the non-fiber beams were failed by shear with a dramatic load drop, regardless of the tensile reinforcement amount, before the yielding of reinforcement and they produced no deflection capability. The test results showed that while the inclusion of steel fibers to the UHPC mixture with low reinforcement ratios changed the failure mode from the shear to flexure, it significantly enhanced the ultimate shear strength in the case of higher reinforcement ratio through the SF-UHPC’ superior mechanical properties and fibers’ crack-bridging ability.

## 1. Introduction

The Steel Fiber-Reinforced Ultra-High Performance Concrete (SF-UHPC) is an advanced type of concrete having the superior mechanical and durability properties. This type of special concrete is used in many areas to eliminate the disadvantages in traditional reinforced concrete member design due to its very high compressive strength and pseudo post-cracking tensile response. While this response can be ensured with a good amount of steel fiber, insufficient fiber volume fraction and/or low concrete compressive strength may lead to softening response [1]. In order to increase the workability of concrete, there is a need to use the superplasticizers through their water reduction ability. It is seen that the new-generation polycarboxylic ether–based superplasticizer can achieve up to 40% water reduction [2,3].

The randomly dispersed steel fibers added to the concrete mixture, which may be in straight, end-hooked, twisted or other forms, improve the capacity in many different ways, such as the ductility, energy absorption capacity and strength. When compared to the conventional normal-strength fibrous concrete (NSFC), the SF-UHPC has very high compressive and tensile strengths, strain capacity and durability through its high-density matrix [4,5,6,7,8]. Hence, the SF-UHPC’s superior characteristics allow the use of higher reinforcement ratios than the limits stipulated in the several design codes.

All material and structural behaviors of the SF-UHPC members are distinct from that of the normal-strength concrete and its use gains many advantages in terms of shear or/and flexural behavior. It is important to note that the current prediction methods of shear strength cannot be directly applied to the SF-UHPC members because of the post-cracking behavior under tension. Regarding the shear behavior of I-shaped SF-UHPC beams, when the principal tensile stress in the web region exceeds tensile stress of concrete, some possible shear failures (such as diagonal tension, shear compression, shear tension, or combination of two or more) come into existence in a very brittle manner depending on the compressive and tensile strengths of concrete, fiber content, shear-span-to-depth (*a*/*d*) ratio and tensile reinforcement ratio. In this sense, the optimum steel fiber content compatible with the reinforcement ratio may be able to replace or reduce the stirrups and/or shear reinforcement with the help of the excellent mechanical properties of the concrete and the crack-bridging ability of steel fibers and hence, formation and propagation of the cracks [9,10,11,12,13,14].

The existing studies on the shear behavior of SF-UHPC can be summarized under two groups. The first group studies were relevant to the importance of steel fiber use on the shear behavior considering the fiber volume fraction, compressive and tensile strengths, shear reinforcement type, tensile reinforcement ratio, *a*/*d* ratio as well as the prestressing conditions. The other group studies focused the feasibility of steel fiber use in place of shear reinforcement. It can be noted that a great majority of these studies intensively focused on the importance of fiber amount on the shear strength and failure mechanisms under the constant tensile reinforcement ratio. In spite of prior valuable studies on the shear behavior, there is a remarkable lack of knowledge about the impact of reinforcement ratio on the SF-UHPC beams’ shear response. Kamal et al. [15] studied the shear behavior of SF-UHPC beams for two tensile reinforcement ratios as well as with and without web reinforcement configurations. Independent of the reinforcement ratio, the steel fibers were more efficient in increasing both initial and ultimate loads. They also indicated that the presence of steel fibers without web reinforcement in the beams was more critical in terms of the ultimate shear strength. El-Dieb et al. [11] investigated the shear response of self-compacting SF-UHPC beams for two fiber volume fractions. The results denoted that the inclusion of steel fibers significantly increased the shear capacity depending on the fiber amount. It was interestingly stated that the effectiveness of steel fiber for the normal- strength concrete was greater than that of the SF-UHPC use. It was also stated that the adding of steel fibers with proper volume fraction could be used in place of the stirrups. Pourbaba et al. [13] studied effects of concrete type, *a*/*d* ratio and reinforcement ratio on the shear behavior. The parametric tests on prism specimens showed that while the normalized shear strength of SF-UHPC beams was averagely 77% greater than those of normal- strength beams, the reinforcement ratio had a limited effect on the shear capacity. Mészöly and Randl [14] revealed that not only the increase of stirrups had less effect on the shear strength rather than both 1 and 2 vol% of fibers, but also the fiber use was more impactful for the case without the shear reinforcement. Ngo et al. [16] conducted a parametric study on prism specimens to investigate the shear strength of SF-UHPC as well as to find a correlation between the shear and tensile strengths. As in other studies, the increased fiber contents enhanced the shear strength. They reported that the shear strength of SF-UHPC beams was particularly influenced by the tensile strength of concrete and *a*/*d* ratio. So the shear strengths of test beams were about 1.6 times greater than the direct tensile strengths, and the *a*/*d* ratio was inversely proportional to the shear strength. This outcome was highlighted at almost all studies considering different *a*/*d* ratios [12,17,18]. Ciprian et al. [19] differently studied the hybrid fiber use in the shear behavior of SF-UHPC beams for different volume fractions. The experimental comparisons pointed out that the hybrid fiber use in the SF-UHPC beams provided better performance, regardless of volumetric fraction, with regard to the ultimate shear strength and deformations compared to the mono fiber. Voo et al. [17] carried out a testing program to investigate the impact of steel fiber content and *a*/*d* ratio on prestressed SF-UHPC beams. The test results showed the shear cracking occurred through the web region prior to the formation of major crack. It was also noted that the fiber content did not significantly affect the cracking load while an increase in volume fraction of steel fiber led to an apparent increase in the shear strength. Baby et al. [20] emphasized an important situation so that even though the use of stirrups in the UHPC beams rather than the steel fiber improved the ultimate shear strength of prestressed and reinforced beams, the association of fiber and shear reinforcement affected them in a negative manner. Baby et al. [10] conducted the material- and structural- scaled shear tests on I-shaped SF-UHPC girders having some types of shear reinforcement with/without the prestressing. The results demonstrated that the shear capacity depended on both the tensile behavior and effect of fiber orientation, prestressing force and cross-sectional geometry.

On the other hand, the feasibility of steel fibers in place of shear reinforcement or shear link on the SF-UHPC members was intensively investigated by some researches. The main outcomes of these studies [15,21,22,23] pointed out that the replacement of shear reinforcement by the steel fibers was applicable to the moderate shear levels and also, the inclusion of steel fiber by 0.75 vol% to the high-strength concrete mixture can be used in place of the minimum shear reinforcement. Zagon et al. [23] investigated the shear behavior of I-shaped SF-UHPC beams with or without web openings. The study reported that the replacement of stirrups by steel fiber was feasible with and without the combination of an additional shear link. However, the test results of eleven T-shaped SF-UHPC beams in Qi et al. [12] showed that the steel fiber use positively affected the post-cracking shear strength and deformability of SF-UHPC beams. Based on the observations, the failure mode of some beams with high reinforcement ratio changed from the shear to shear-flexure. This indicates that the fibers are more impactful in enhancing the shear strength than flexural strength. Yavas et al. [9] investigated the effects of different fiber type and amounts on the shear behavior. While the steel fiber use has no notable impact on the cracking load for low volume fractions, regardless of fiber type, the cracking load shows an increasing trend for high fiber volume fractions. The test results also indicated the applicability of steel fiber as the shear reinforcement. Hasgul et al. [24] recently studied the feasibility of steel fibers in place of shear reinforcement in the I-shaped SF-UHPC beams. Even though no shear reinforcement is provided in the test beams in contrast with the requirements in design codes, the inclusion of steel fibers of 1.5 vol% is sufficient to guarantee the flexural behavior.

The aim of this experimental study is to investigate the impacts of steel fiber use and tensile reinforcement ratio on the shear behavior of I-shaped SF-UHPC beams. In this sense, the total of eight beams consisting of four reinforcement ratios (in a range of 0.8% to 2.2%) were casted without shear reinforcement and subjected to the four-point loading test. While half of the test beams included 30 mm end-hooked steel fibers with 2.0 vol%, the remaining beams were produced without the fiber to show possible effectiveness of the fiber use. Based on the non-fiber beam configurations, the shear performances of SF-UHPC beams were discussed in terms of the load-deflection response, cracking pattern, first cracking strength and ultimate shear strength. In addition, the well-known code equations (ACI 318 [25], Eurocode 2 [26], NZS 3101 [27] and Model Code 2010 [28]) in relation to the nominal shear capacity were discussed by referring to the experimental results of the non-fiber reference beams. In the second part of numerical investigations, the flexural capacity of test beams except for the shear dominant beams were numerically predicted and compared with the experimental results.

## 2. Experimental Program

### 2.1. Material Properties and Mixture Design

In the study, two concrete mixtures were designed with and without steel fiber. Here, the first one is the plane mixture without the steel fiber, which can be classified as the Ultra High Performance Concrete (UHPC), the second one is the SF-UHPC mixture showing the post-cracking behavior under bending thanks to the steel fibers. In the plain mixture, Portland cement (*C*), silica fume (*SF*) and blast-furnace slag (*BFS*) were used as the binder part of concrete. Two different sizes of quartz sand (*QS*) were combined for the aggregate. A very low water-to-binder ratio (*W*/*B*) of 0.18 was used for both concrete mixtures. In order to ensure the adequate workability for the considered *W*/*B* ratio, the polycarboxylate ether superplasticizer (*PCE*) was used as a high range water reducing admixture. In the SF-UHPC mixture, the end-hooked steel fiber of 30 mm long and 0.55 mm in diameter were used of 2.0 vol%. The specified tensile strength of fibers was 1345 MPa. The material quantities in 1 m^3^ volume by weight are presented for each concrete mixture in Table 1. The sizes, densities and surfaces of considered materials can be found in the references [7] and [9].

**Figure 1:**
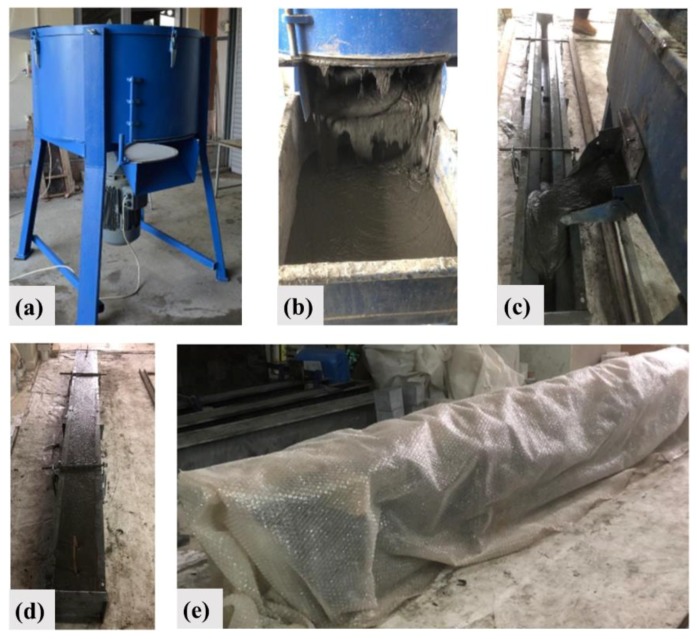
(**a**) Horizontal pan mixer, (**b**) Pouring, (**c**) Casting process, (**d**) After casting, (**e**) Covered beam.

The casting stages are summarized in Figure 1a–d. A horizontal pan mixer was used for the preparation of all concrete mixtures, and it allows different rotational speeds (Figure 1a). Firstly, all the binder materials and aggregates were dry mixed for about three minutes with the help of a pan mixer. Later on, all of the water and half of the plasticizer were added to the dry mixture and were mixed for at least another three minutes. The remaining plasticizer was slowly introduced to the mixture of plastic consistency. Thus, the sufficient fluidity was ensured after approximately five more minutes (Figure 1b). Finally, the steel fibers, if any, were added gradually to the mixture, and the mixing procedure was terminated after a stable and homogenous form was obtained within an average of ten minutes. A specially designed rail pouring car was used for the concrete casting (Figure 1c). In order to ensure the fiber orientation in the longitudinal direction of beam, the pouring was carried out at several stages by moving from one end of the beam mold to the other, as shown in Figure 1c. The mixtures have largely self-compacting characteristic and no vibration was applied during casting to avoid the fiber gravitation. After the casting stage, the beams were covered with a plastic sheet (Figure 1d,e) to prevent the surface evaporation and autogenous shrinkage cracks. The specimens were demolded after 24 h and stored in the laboratory at ambient temperature until the test day. It should be noted that the plastic sheet was removed after the demolding.

### 2.2. Material Based and Resistant Features

The mechanical properties of UHPC and SF-UHPC mixtures were evaluated by means of the compressive, tensile and flexural responses. In determination of the compressive strength (*f_c_*’) in relation to each mixture, six cubic specimens with 100 mm edge were prepared and subjected to the uni-axial compression tests [29]. The tests were conducted using a 3000 kN load capacity hydraulic press under the loading rate of 1 MPa/s.

**Figure 2:**
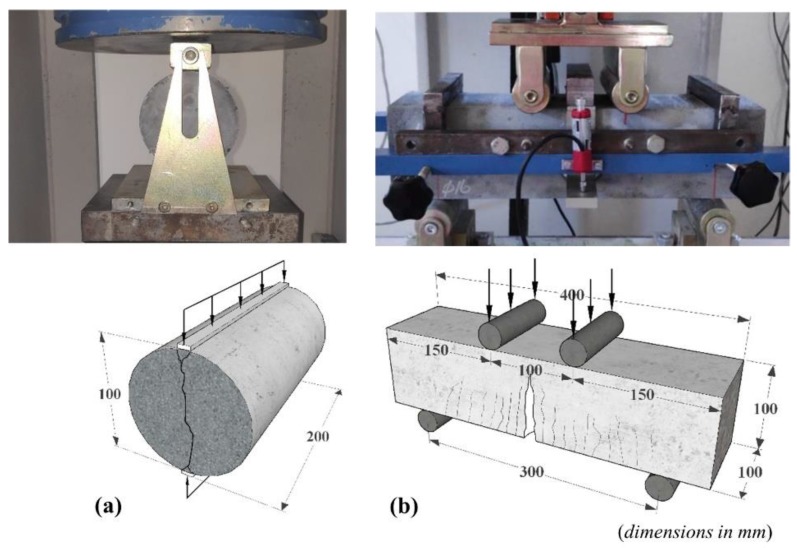
(**a**) Splitting tensile test setup, (**b**) Flexural test setup.

The tensile strengths with respect to the mixtures were obtained from the splitting tensile tests in accordance with the ASTM C 496 [30]. The six cylinder specimens were prepared with 100 mm diameter and 200 mm height for each mixture. By means of the hydraulic press, the compressive load was applied to the diametrical along the transverse direction of specimen (Figure 2a). After that, the splitting tensile strength (*f_sp_*) of the specimens was calculated by Equation (1), where the *P* is the maximum applied load, *l* and *D* are the length and diameter, respectively.

Not only to show the deflection hardening characterization in relation to the mixtures, but also to show the energy absorption capacity of material, the flexural tests were performed on simply supported prismatic specimens with a square section of 100 × 100 mm^2^ and length of 400 mm in accordance with the ASTM C 1609 standard [31]. The geometric properties of test specimen and loading scheme are presented in Figure 2b. The average mid-span deflections were determined from two linear variable displacement transducers (LVDT) fixed on both sides of the specimen. In this way, the effect of possible settlements at the supports were eliminated (Figure 2b). The tests were conducted under the displacement control using the servo-hydraulic machine with 500 kN capacity. The load increment rate for the deflection was taken as 0.02 mm/min. The 24-channel data acquisition system, with the sampling rate of 8 Hz per channel, was used to record the signals related to the load and deflection. The flexural performances of UHPC and SF-UHPC mixtures were discussed by the flexural strength (*f_p_*) and toughness values (energy dissipation capacity). The strengths were also calculated by Equation (2) in the ASTM C 1609. In Equation (2), *P* denotes the applied load, *b*, *d* and *L* are the width, depth and span length of test specimen, respectively. In calculation of the toughness values (*T*) in relation to the total area under the load-deflection curve, the deflection limit of *L*/150 is based on [31].

(1) $$f_{sp}=\frac{2P}{\pi lD}$$

(2) $$f_{p}=\frac{PL}{bd^{2}}$$

### 2.3. Preparition of Test Beams and Four-Point Loading Tests

In the experimental program, four tensile reinforcement ratios representing low to high ratios ranged from 0.8% to 2.2% were considered to discuss different shear demands of the UHPC beams with/without the steel fiber. In this sense, the total of eight simply supported reinforced concrete beams, in which half of the beams were made from the SF-UHPC and the remaining beams were produced without the fiber to show the possible effectiveness of fiber use, were casted and subjected to the four-point loading test.

**Figure 3:**
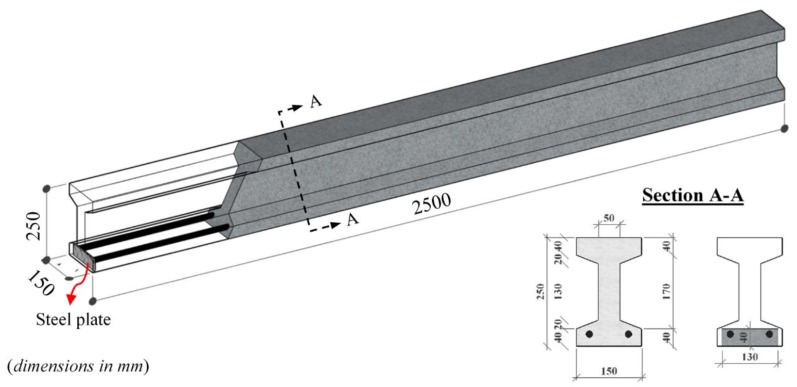
A typical singly reinforced concrete test beams.

The I-shaped beam cross-sections comprised of 150 mm flange width, 60 mm flange thickness, 50 mm web thickness and 250 mm height. The total length of beams is 2500 mm, as shown in Figure 3. The singly reinforced beams have several sizes of deformed reinforcing steel bars. However, all beams had the concrete cover of 20 mm and hence, effective depths became variable. The tensile reinforcements and mixture types are presented in Table 2. It is important to note that the considered reinforcement ratio of 2.2% almost corresponds to the upper limits stipulated in the several design codes such as, TS-500 [32], ACI 318 [25] and Eurocode-2 [26]. The yield and ultimate strengths of tensile reinforcements were determined by means of the uni-axial tension tests on the reinforcement coupons of 300 mm [33], and the average values of 470 and 593 MPa were determined, respectively. In Table 2, the notations *P* and *F* denote the absence or presence of steel fiber, respectively. The reinforcement ratios were added to the beam codes as a percentage. For instance, while the code F-1.7 denotes the test beam including the tensile reinforcement ratio of 1.7% and the end-hooked steel fibers of 2.0 vol%, the P-1.7 indicates the plain beam with same reinforcement ratio. In order to prevent pullout in the beams, the both ends of tensile reinforcements were welded by a steel plate. No shear reinforcement was placed to the test beams to show the potential advantages of fiber use.

**Figure 4:**
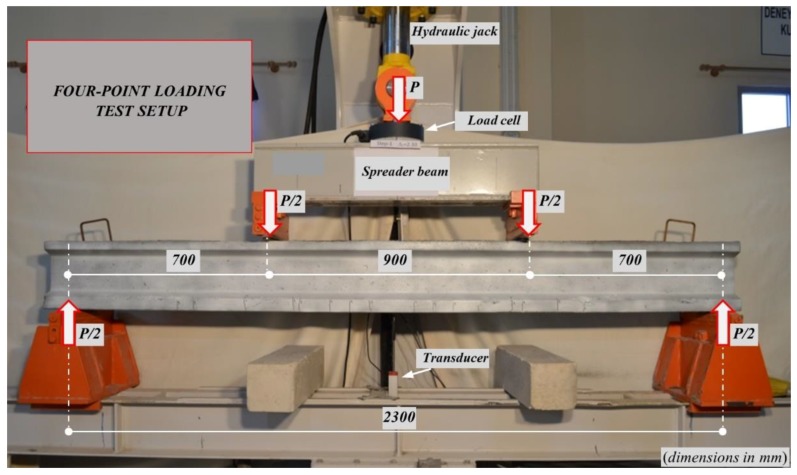
The four-point loading setup.

The four-point loading scheme results in a shear-span of 700 mm as shown in Figure 4. The *a*/*d* ratio was chosen larger than 2.5 (roundly 3.1) in order to reduce any contribution from arch action to the shear strength for all beams. A load cell with 500 kN capacity was placed on the actuator to measure the applied load (*P*). The *P* was transferred to the test beam as two-point loads of *P*/2 by means of a steel spreader beam (Figure 4). The mid-span deflections were determined from the potentiometric transducer with the capacity of 250 mm. All measurements were recorded simultaneously by using a data logger system with the 24-channel and sampling rate of 8 Hz per channel.

## 3. Test Results and Discussions

### 3.1. Material Test Results

The results from six cubic and cylinder specimens for each mixture were averaged to determine the compressive and tensile strengths (Table 3). While the steel fiber use increased the compressive strength by 18% in comparison to the non-fiber mixture, it enhanced the tensile strength 3 times. Here, the impact of fiber use became more prominent on the tensile behavior.

**Figure 5:**
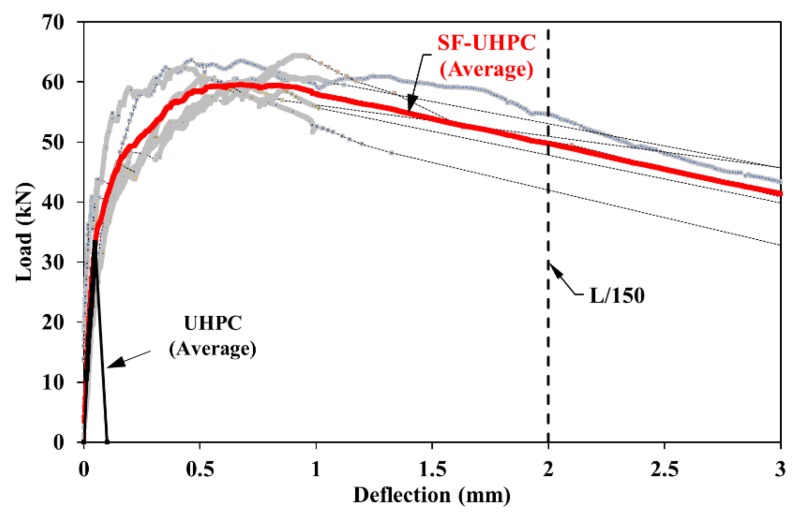
Load-deflection curves of prismatic specimens with and without steel fiber.

The load-deflection curves obtained from the four-point loadings of prismatic specimens and their average responses are illustrated in Figure 5. While the SF-UHPC specimens exhibited a higher load-carrying capacity after the first crack, which characterizes the deflection hardening response in the flexure, the non-fiber specimens failed without providing any additional capacity just after cracking. It can be clearly seen from Figure 5 that the steel fibers not only increased the load capacity, but also allowed large deflection capability after the peak load. The average flexural strength of SF-UHPC was obtained 79% higher rather than the non-fiber mixture. It was observed that when the ultimate deflection of *L*/150 was reached, an evident amount of strength loss could be seen on the load-deflection curve. It should be noted that the steel fiber use extremely increased the deflection capacity and hence, the energy dissipation capacity extremely improved (Table 3).

Based on the material test results, the inclusion of steel fibers to the UHPC mixture considerably improved the mechanical properties of concrete. The main reason is that the steel fibers inhibited the crack propagation and width through the fibers’ crack-bridging ability [9,10,11,12,13,14]. Consequently, the fiber use considered in the volume fraction provided superior compressive and tensile strengths as well as the flexural responses in comparison to the non-fiber configuration.

### 3.2. Structural Test Results

#### 3.2.1. Load-Deflection Relationships

**Figure 6:**
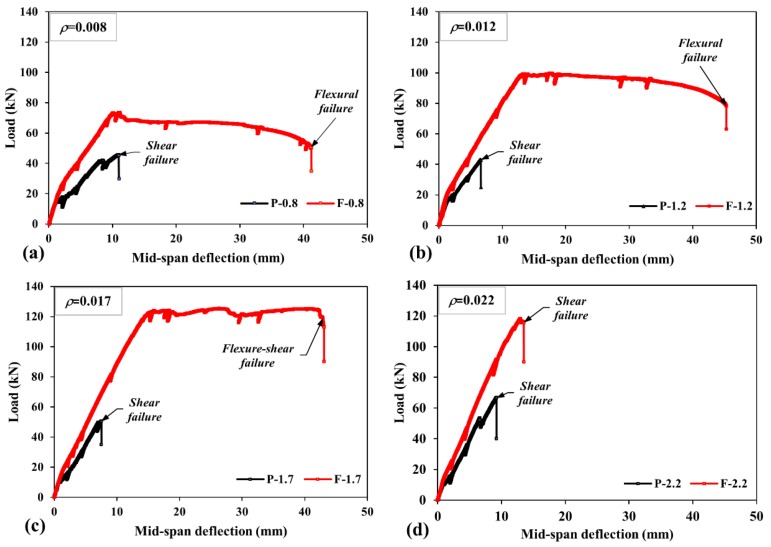
Load-deflection curves of test beams, (**a**) *ρ* = 0.008, (**b**) *ρ* = 0.012, (**c**) *ρ* = 0.017, (**d**) *ρ* = 0.022.

Figure 6 shows the measured load—deflection responses for each reinforcement ratio. Noted that all test beams exhibited almost linear behavior until the first crack occurrence. Later on, the slope of curves and hence, lateral stiffness reduced. After this point, even though all UHPC beams failed by the shear before the yielding of reinforcement without any deflection capability, two responses for the SF-UHPC beams showed up according to the failure type as the shear or flexure (Figure 6 and Table 4).

#### 3.2.2. Failure Modes and Cracking Patterns

**Figure 7:**
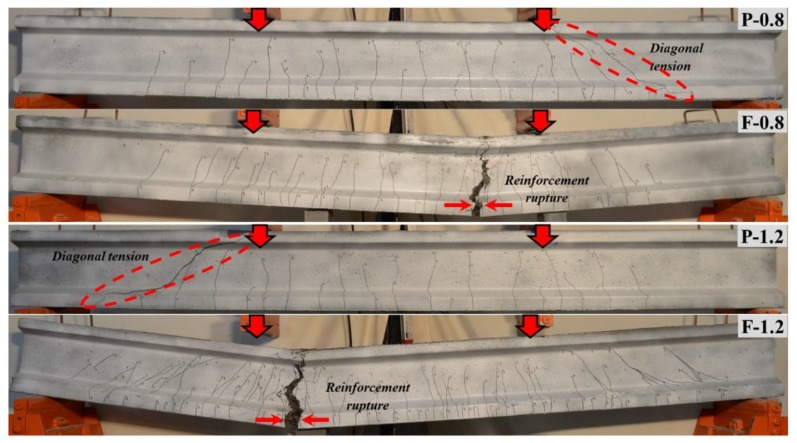
Comparison of failure modes for the test beams with low reinforcement ratios.

**Figure 8:**
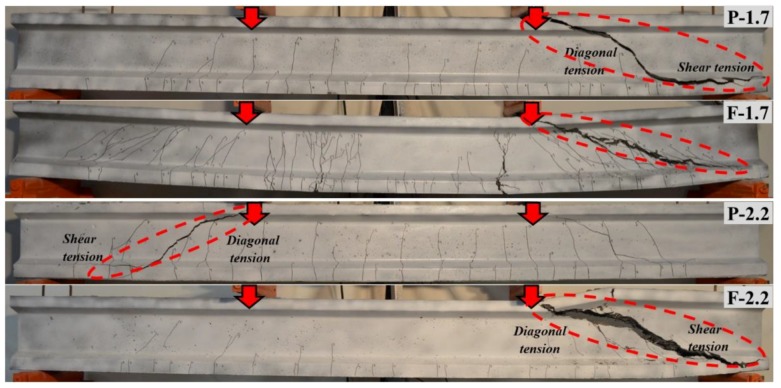
Comparison of failure modes for the test beams with high reinforcement ratios.

The comparisons regarding the failure modes of beams for each reinforcement ratio are shown in Figure 7 and Figure 8. All UHPC beams, which did not include the steel fibers and shear reinforcement, behaved in a similar manner with multiple vertical cracks formed at first in the constant-moment region and extended towards to the compression zone. Later on, the governing diagonal crack showed up in the mid-depth of the web region. Immediately afterwards, it rapidly propagated towards the support and the loading point. Finally, the beams failed by shear after this crack formation (Figure 7). This type of failure can be described as the diagonal tension (DT) failure. However, the DT failure of some UHPC beams (P-1.7, P-2.2 and F-2.2) with high reinforcement ratios combined with the shear tension (ST) where the diagonal crack propagated along the longitudinal reinforcement level resulting from the bond loss, as shown in Figure 8.

Referring to Figure 7, the addition of steel fibers was able to prevent the shear failure without need to the shear reinforcement for SF-UHPC beams with low reinforcement ratios (F-0.8 and F-1.2). In this response, the fibers particularly prevented the crack formation as well as restricted the propagation of diagonal cracks due to their bridging ability. It should be also noted that only well disturbed flexural cracks propagated towards the compression zone (Figure 7). After with further load increment, the deformation of tensile reinforcement with respect to these beams concentrated at the point where one major crack widened. This crack width intensely enlarged against other cracks due to the crack localization phenomenon, as shown in Figure 7. Hence, the reinforcement ruptured (RR) prior to the concrete crushing in the compression region. It should be noted that this event may also occur in structural member including normal- and high- strength fibrous concretes. As parallel to this outcome, some studies pointed out the crack localization and its negative effect on the flexural ductility for very low reinforcement ratios [6,7,34,35,36,37,38,39,40].

At high reinforcement ratios of 1.7% and 2.2%, the fibers were insufficient to prevent the shear failure of the SF-UHPC beams. However, it should be clarified that the beam F-1.7 resulted in a mixed failure mode as the flexure-shear where a diagonal crack was accompanied by the concrete crushing after the yielding condition (Figure 8). Unlike the UHPC beam, many fine inclined cracks occurred in the beams F-1.7 close to the main diagonal crack which indicates the bridging ability of randomly dispersed fibers. This beam also reached to remarkable flexural capacity before the shear failure. When the normal-strength concrete instead of the UHPC was considered, such a deflection ductility wouldn’t be possible because of higher tensile reinforcement ratio. The fiber use provided exceptional contribution to the SF-UHPC beams without the shear reinforcement. However, the fibers could not prevent the shear failure for the beam F-2.2. Even so, the fibers delayed the formation of governing shear crack and hence, the shear strength increased about 2 times in comparison to the non-fiber condition.

#### 3.2.3. The First Cracking Load

For the considered test beams, the initial cracking loads were determined based on the point at which the first slope changes in the load-deflection curve. In order to discuss the impacts of fiber use and reinforcement ratio on the cracking behavior, the cracking loads (*P_cr_*) and corresponding deflections (Δ*_cr_*) are listed in Table 4. Regardless of the fiber presence in the test beams, the cracking loads have a decreasing trend as the reinforcement ratio increases. While the first cracking points with respect to the beams P-0.8 and F-0.8 with the lowest reinforcement ratio developed at the loads of 15.43 kN and 24.77 kN, respectively, in conjunction with the highest reinforcement ratio of 2.2%, these values decreased to 10.28 kN and 13.38 kN. It should be noted that even though the first cracking load is expected to slightly increase as the reinforcement ratio increases for the conventional concrete, this behavior has a reverse situation for the UHPC members due to the dense matrix as well as very high bond strength between the fiber and concrete matrix. However, all the SF-UHPC beams had notably higher cracking load than the UHPC beams. In this sense, the fiber use increased the cracking load up to 1.6 times depending upon the reinforcement ratio. This proves that the steel fiber use has a remarkable influence on the cracking behavior of SF-UHPC beams with the help of the crack-bridging ability.

#### 3.2.4. Ultimate Shear Capacity

In order to show the influences of fiber presence and different reinforcement ratios on the shear capacity, the ultimate shear strengths (*V_u_*) of test beams are presented in Table 4. Here, the *V_u_* was taken as half of the peak load (*P_p_*) obtained in the shear dominant beams (Table 4). Referring to Table 4, the shear strength of UHPC beams showed, in general, an increasing trend with the increase of tensile reinforcement ratio. While the beam P-0.8 with the lowest reinforcement ratio had the shear strength of 22.85 kN, the P-2.2 exhibited 1.5 times more shear resistance. Since the tensile strength of concrete is one of the most influential features in terms of the shear strength, the capacities of SF-UHPC beams extremely improved through the crack-restriction ability of fibers. Accordingly, the shear strengths of 62.65 kN and 60.09 kN were determined for the SF-UHPC beams having the reinforcement ratios of 1.7% and 2.2%, respectively. We want to draw attention to the fact that the steel fiber use increased the shear strength by 1.8 and 2.5 times in comparison to the plain configuration with the same reinforcement ratio.

## 4. Numerical Predictions of the Shear and Flexural Capacities

Regarding the nominal shear capacity of normal- and high-strength reinforced concrete beams, some equations were stipulated in well-known design codes such as, ACI 318 [25], Eurocode 2 [26], NZS 3101 [27] and Model Code 2010 [28]. These equations can be applied up to an upper limit of compressive strength of 90 MPa. However, it is unknown whether or not these practical equations are applicable for the beams made from the non-fiber UHPC. In the current study, the nominal shear capacities of UHPC beams (P-0.8, P-1.2, P-1.7 and P-2.2) without the shear reinforcement were calculated by different code equations, and the results were discussed by referencing the experimental capacities.

On the other hand, although some recommendations [1,41,42,43] were presented with regard to the flexural moment capacity of the SF-UHPC members, these approaches have not been included in a design code yet. Many efforts were conducted to practically predict the capacities of SF-UHPC beams investigating the shape of concrete stress block, ultimate strain capacity, fibers’ geometrical properties, volumetric ratio, orientation, bond stress as well as other parameters affecting the tensile stress distribution [44,45,46,47,48,49,50,51,52]. In the second part of numerical investigation, the flexural capacities of test beams, except the shear dominant beams, were numerically predicted based on the model proposed in ACI 544 code [53] and adaptation in Imam et al. [48] for the calculation of equivalent concrete tensile strength. The numerical predictions were compared and discussed with the experimental results.

### 4.1. Prediction of the Nominal Shear Strengths

As noted above, the nominal shear strengths of all UHPC beams without the shear reinforcement were calculated by means of Equations (3)–(11), which are given in the ACI 318, Eurocode 2, NZS 3101 and Model Code 2010, respectively.

In Equation (3), *ρ* denotes the longitudinal reinforcement ratio; *a*, *d* and *b_w_* are the shear span, effective depth and width of beam, respectively [33]. In Equation (4), *k* is the aggregate size factor (= 1 + (200/*d*)^0.5^) [34]. In Equations (5) and (6), *k_d_* represents the influence of member depth on the shear strength. If the *d* ≤ 400 mm, *k_d_* = 1.0; *d* > 400 mm, *k_d_* = (400/*d*)^0.25^. *k_a_* is an impact factor for the maximum aggregate size (*d_a_*). If the *d_a_* ≥ 20 mm, *k_a_* = 1.0; *d_a_* ≤ 10 mm, *k_a_* = 0.85. Note that the *d* should be in mm in calculation of the coefficients *k* and *k_d_*. The value of *v_c_* can be calculated by linear interpolation of 200 mm < d <400 mm [35]. In the Model Code 2010, the level III approximation was considered for calculating the shear resistance attributed to the concrete (*V_Rd,c_*) (Equation (8)). In Equations (8)–(11), *z* is the effective shear depth, *γ_c_* is the partial safety factor for concrete and *k_c_* denotes the strength reduction factor. The *k_v_* value is calculated by Equation (9) and includes the longitudinal strain (*ε_x_*) in the mid-depth at the control section by using Equation (10). The limit of shear strength (*V_Rd,max_*) is calculated by Equation (11) to prevent the premature concrete crushing. *M_Ed_* and *V_Ed_* are the design moment and shear force in the control section, respectively, *E_s_* and *A_s_* represent the modulus of elasticity and area of the longitudinal reinforcement. Here, the inclination of compressive stress field (*θ*) should be between 20° + 10,000*ε_x_* and 45° [28].

(3) ACI 318 : Vn=[fc′+120ρ(da)]bwd7 (MPa)

(4) $$\mathrm{Eurocode}\;2\;:\;V_{Rd,c}=\left[0.18k{\left(100\rho f_{c}\prime \right)}^{1/3}\right]b_{w}d\;\left(MPa\right)$$

(5) $$\mathrm{NZS}\;3101\;:\;V_{c}=k_{d}k_{a}v_{b}b_{w}d\;\;\;d\geq 400\;mm\;\left(MPa\right)$$

(6) $$V_{c}=max\left[Eq.\left(3a\right);0.17k_{a}\sqrt{f_{c}\prime }b_{w}d\;\right]\;d\leq 200\;mm$$

(7) $$v_{b}=min\left[\left(0.07+10\rho \right)\sqrt{f_{c}\prime }\;;0.2\sqrt{f_{c}\prime }\;\right]\geq 0.08\sqrt{f_{c}\prime }$$

(8) $$\mathrm{Model}\;\mathrm{Code}\;2010\;:\;V_{Rd,c}=k_{v}\left(\sqrt{f_{c}\prime }/\gamma_{c}\right)zb_{w}$$

(9) $$k_{v}=\frac{0.4}{\left(1+1500\varepsilon_{x}\right)}\left[1-\frac{V_{Ed}}{V_{Rd,max}\left(\theta_{min}\right)}\right]\geq 0$$

(10) $$\varepsilon_{x}=\left(M_{Ed}/z+V_{Ed}\right)/\left(2E_{s}A_{s}\right)$$

(11) $$V_{Rd,max}=k_{c}\frac{f_{c}\prime }{\gamma_{c}}b_{w}z\sin\theta \cos\theta$$

Referring to Table 5, the calculated shear capacities (*V_n_^cal^*) were proportioned to the experimental capacities (*V_n_^exp^*) to show the performances with respect to the code equations. It can be deduced that all code equations conservatively predicted the shear strength with the errors varying in range of 4% to 54%. However, the relative error for the highest reinforcement ratio is more apparent. While the best predictions among all approaches were obtained by the Eurocode 2 equation with the errors varying ranged from 4% to 24%, the worst results were obtained from the Model Code 2010. It was noted that the error rates for other three approaches increased up to 55%, especially at the highest reinforcement ratio. Although the considered code equations were in different forms, all of them gave close predictions especially for the beams P-0.8 and P-1.2. It is seen from the results that unlike the conventional concrete, considering the features such as very small aggregate diameter and very high compressive strength, these approaches should be improved to apply in the UHPC members. It can be also seen from the limited results obtained from the study that the effect of reinforcement ratio on shear strength for this high class of concrete, which allows the use of high reinforcement ratios under flexure, should be investigated.

### 4.2. Prediction of the Flexural Moment Capacities

**Figure 9:**
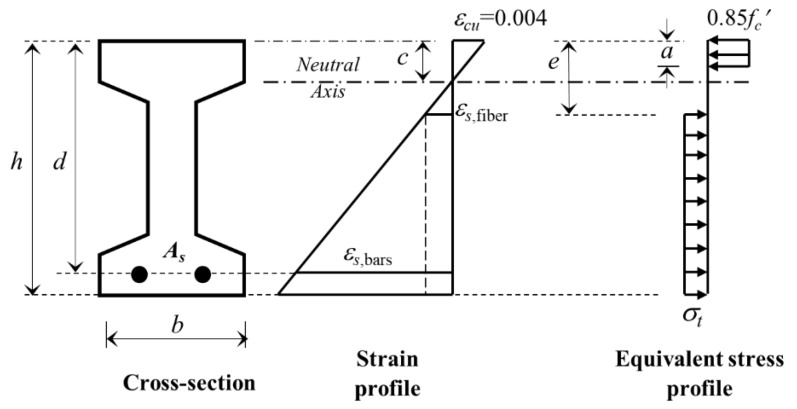
The strain and stress distributions relation to numerical model [53].

The flexural design principles developed for the normal-strength fibrous concretes cannot be directly applied to the SF-UHPC members since the compressive and tensile responses are distinctive due to the strength and post-cracking behavior. In the second part of analyses, the moment capacities of SF-UHPC beams showing the flexural behavior were predicted by using the approaches proposed in ACI 544 [53] and Imam et al. [48]. Working with this model, the use of well-known equivalent stress block was maintained for the compression and tension regions, as shown in Figure 9.

The ACI 544 [53] proposed a numerical model to predict the nominal moment capacity of singly reinforced fibrous concrete beams. The equivalent tensile strength (*σ_t_*) is calculated by Equation (12). It is assumed that the yield condition of tensile reinforcement governed the ultimate capacity. Equation (12) is essentially based on the fiber bond strength (*τ_f_*) of 2.3 MPa for the normal-strength concrete. However, this value for high strength fibrous concretes should be greater than that of the normal-strength concrete due to their dense matrix. Naaman and Najm [54] stated that the bond strength value is in a range of 1–9 MPa depending upon the concrete compressive strength and fiber characteristics. For high strength concrete, Imam et al. [48] used the bond strength value of 4.15 MPa. Accordingly, the Equation (12) was transformed to Equation (13). In the Equations (12) and (13), (*l_f_* / *d_f_*) denote the fiber aspect ratio, *ρ_f_* is the volumetric fiber ratio and *F_be_* is the bond efficiency of fibers.

(12) $$\sigma_{t}=0.00772\left(\frac{l_{f}}{d_{f}}\right)\rho_{f}F_{be}$$

(13) $$\sigma_{t}=0.02\left(\frac{l_{f}}{d_{f}}\right)\rho_{f}F_{be}$$

The Equation (13) was applied to the test beams showing the flexural behavior, and the calculated moment capacities (*M_p_^cal^*) were proportioned to the experimental capacities (*M_p_^exp^*), as shown in Table 6. It is apparent that the moment predictions for considered SF-UHPC beams are highly compatible with the test results, regardless of the reinforcement ratio. Referring to Table 6, the errors are in a small band less than 5%. It can be deduced that the practical approach can satisfactorily determine the flexural moment capacities of SF-UHPC beams.

## 5. Conclusions

In the presented study, the impacts of steel fiber use and tensile reinforcement ratio on the shear behavior was experimentally investigated on I-shaped UHPC beams. In this sense, the total of eight beams consisting of four reinforcement ratios (ranging from 0.8% to 2.2%) were casted without shear reinforcement and subjected to the four-point loading test. The shear performances were discussed in terms of the load-deflection response, cracking pattern, first cracking strength and ultimate shear strength. In addition, the nominal shear and flexural capacities of test beams were numerically predicted and separately discussed with the experimental results. The following outcomes can be deduced from the experimental and numerical investigations:

Based on the material test results, the inclusion of steel fibers to the UHPC mixture considerably improved the mechanical properties of concrete. The main reason is that the steel fibers inhibited the crack propagation and width through the fibers’ crack-bridging ability. However, the steel use in the considered volume fraction provided the superior compressive and tensile strengths as well as the flexural responses in comparison to the non-fiber configuration. It can be also noted that the impact of fiber use became more prominent on the tensile behavior.

Whereas all UHPC beams failed by the shear before the yielding of reinforcement without any deflection capability, two responses for the SF-UHPC beams showed up according to the failure type as the shear or flexure. The addition of steel fibers was able to prevent the shear failure without need to the shear reinforcement for SF-UHPC beams with low reinforcement ratios since the fibers particularly prevented the crack formation as well as restricted the propagation of diagonal cracks due to their bridging ability. However, at high reinforcement ratios of 1.7% and 2.2%, the fibers were insufficient to prevent the shear failure of the SF-UHPC beams. However, the fibers delayed the formation of shear crack and, hence, the shear strength increased about 2 times in comparison to the non-fiber condition.

Regardless of the fiber presence in the test beams, the cracking load has a decreasing trend as the reinforcement ratio increases. However, the SF-UHPC beams had notably higher cracking load than the UHPC beams. This proves that the steel fiber use had a remarkable influence on the cracking behavior of SF-UHPC beams. From the point view of the shear strength, an increasing trend was determined with the increasing of tensile reinforcement ratio. This observation is very similar to those reported in research by others [13,55,56]. The fiber use increased the shear strength by 1.8 and 2.5 times in comparison to the plain configuration with same reinforcement ratio.

When the numerical predictions for the non-fiber beams were discussed, the ACI 318, Eurocode 2, NZS 3101 and Model Code 2010 equations conservatively determined the shear strength with the errors in the range of 4% to 55%. However, the relative error is more apparent for the highest reinforcement ratio. It is thought that these equations should be improved by considering the specific properties of mixture, very high compressive strength as well as different *a*/*d* and the reinforcement ratios. The moment predictions of considered SF-UHPC beams are highly compatible with the test results, regardless of the reinforcement ratio. It should be highlighted that the errors are in a small band less than 5%. It can be deduced that the practical approach can satisfactorily determine the flexural moment capacities of SF-UHPC beams.

One of the promising methods to improve the shear and flexural performances of SF-UHPC members is to blend together two or more steel fiber types in a matrix since the micro and macro steel fibers play a role at two different levels depending upon the fiber length. In the hybrid fiber reinforced concrete, while the micro fibers improve the strength and stiffness in the pre-peak area since the crack widths are still tight, the macro fibers limited the major cracks and its propagation. The investigation of hybrid fiber use on mechanical properties of the SF-UHPC will provide remarkable knowledge and, hence, it has potential to change the mono fiber use by the multiple use.

## Figures and Tables

**Table 1:** Mixture proportions per unit weight (kg/m^3^).

Mixture	C	SF	BFS	QS1 (0–0.8 mm)	QS2 (1–3 mm)	Water	PCE	Steel Fiber
UHPC	690	138	276	542	542	199	17.25	—
SF-UHPC	690	138	276	535	535	199	17.25	2.0 vol%

**Table 2:** Reinforcement and concrete contents of test beams.

Beam	Tensile Reinforcement	Reinforcement Ratio (*ρ*)	Concrete Mixture	Steel Fiber
P-0.8	2ϕ10	0.8%	UHPC	—
P-1.2	2ϕ12	1.2%		
P-1.7	2ϕ14	1.7%		
P-2.2	2ϕ16	2.2%		
F-0.8	2ϕ10	0.8%	SF-UHPC	2.0 vol%
F-1.2	2ϕ12	1.2%		
F-1.7	2ϕ14	1.7%		
F-2.2	2ϕ16	2.2%		

**Table 3:** Mechanical properties of the mixtures.

Mixture	*f_c_*′ (MPa)		*f_sp_* (MPa)		*f_p_* (MPa)		*T* (kNmm)	
	Mean	Std	Mean	Std	Mean	Std	Mean	Std
UHPC	121	1.8	5.70	0.15	9.98	0.33	1.71	0.04
SF-UHPC	143	1.9	17.10	0.21	17.88	0.63	152.9	11.06

**Table 4:** The main deflection and load results.

Beam	Δ*_cr_* (mm)	*P_cr_* (kN)	Δ*_p_*(mm)	*P_p_* (kN)	*V_u_* (kN)	Failure Mode	
P-0.8	1.21	15.43	10.92	45.69	22.85	Shear	(DT)
P-1.2	1.23	15.62	6.52	43.12	21.56	Shear	(DT)
P-1.7	0.76	9.96	7.54	50.60	25.30	Shear	(DT + ST)
P-2.2	0.85	10.28	9.22	66.81	33.40	Shear	(DT + ST)
F-0.8	1.98	24.77	11.01	73.52	—	Flexure	(RR)
F-1.2	1.26	19.78	17.56	99.78	—	Flexure	(RR)
F-1.7	1.22	16.03	26.33	125.31	62.65	Flexure shear	(DT + ST)
F-2.2	0.89	13.38	12.97	120.18	60.09	Shear	(DT + ST)

Δ_cr_: Cracking deflection, P_cr_: Cracking load, Δ_p_: Peak deflection, P_p_: Peak load, V_u_: Shear capacity. DT: Diagonal tension, (DT + ST): Combination of diagonal tension and shear tension, RR: Reinforcement rupture.

**Table 5:** Nominal shear strengths of ultra-high performance concrete (UHPC) beams.

Beam	Design Code	*V_n_^exp^* (kN)	*V_n_^cal^* (kN)	*V_n_^cal^*/*V_n_^exp^*
P-0.8	ACI 318	22.85	18.18	0.80
	Eurocode 2	22.85	18.06	0.79
	NZS 3101	22.85	17.62	0.77
	Model Code	22.85	13.10	0.57
P-1.2	ACI 318	21.56	18.43	0.85
	Eurocode 2	21.56	20.68	0.96
	NZS 3101	21.56	18.14	0.84
	Model Code	21.56	15.81	0.73
P-1.7	ACI 318	25.30	18.74	0.74
	Eurocode 2	25.30	23.22	0.92
	NZS 3101	25.30	18.28	0.72
	Model Code	25.30	16.23	0.64
P-2.2	ACI 318	33.40	19.05	0.57
	Eurocode 2	33.40	25.31	0.76
	NZS 3101	33.40	18.28	0.55
	Model Code	33.40	15.05	0.45

**Table 6:** Numerical analysis results of the Steel Fiber-Reinforced (SF)-UHPC beams.

Beam	*M_p_^exp^* (kNm)	*M_p_^cal^* (kNm)	*M_p_^cal^*/*M_p_^exp^*
F-0.8	25.7	26.2	1.02
F-1.2	34.9	33.1	0.95
F-1.8	43.8	41.1	0.94

## References

[1] Russell H.G., Graybeal B.A. (2013). Ultra-High Performance Concrete: A State-of-the-Art Report for the Bridge Community.

[2] Li P.P., Yu Q.L., Brouwers H.J.H. (2017). Effect of PCE-type superplasticizer on early-age behaviour of ultra-high performance concrete (UHPC). Constr. Build. Mater..

[3] Zhang Y., Kong X. (2015). Correlations of the dispersing capability of NSF and PCE types of superplasticizer and their impacts on cement hydration with the adsorption in fresh cement pastes. Cem. Concr. Res..

[4] Wille K., Naaman A.E., El-Tawil S., Parra-Montesinos G.J. (2012). Ultra-high performance concrete and fiber reinforced concrete: Achieving strength and ductility without heat curing. Mater. Struct..

[5] Kim D.J., Park S.H., Ryu G.S., Koh K.T. (2011). Comparative flexural behavior of hybrid ultra high performance fiber reinforced concrete with different macro fibers. Constr. Build. Mater..

[6] Turker K., Hasgul U., Birol T., Yavas A., Yazici H. (2019). Hybrid fiber use on flexural behavior of ultra high performance fiber reinforced concrete beams. Compos. Struct..

[7] Hasgul U., Turker K., Birol T., Yavas A. (2018). Flexural behavior of ultra-high-performance fiber reinforced concrete beams with low and high reinforcement ratios. Struct. Concr..

[8] Turker K., Birol T., Yavas A., Hasgul U., Yazici H. (2019). Flexural behavior of beams with ultra high performance fiber reinforced concrete. Tech. J..

[9] Yavas A., Hasgul U., Turker K., Birol T. (2019). Effective fiber type investigation on the shear behavior of ultrahigh-performance fiber-reinforced concrete beams. Adv. Struct. Eng..

[10] Baby F., Marchand P., Toutlemonde F. (2014). Shear behavior of ultrahigh performance fiber-reinforced concrete beams. I: Experimental investigation. J. Struct. Eng..

[11] El-Dieb A.S., El-Maaddawy T.A., Al-Rawashdah O. Shear Behavior of Ultra-High-Strength Steel Fiber Reinforced Self-Compacting Concrete Beams. Proceedings of the First International Conference on Construction Materials and Structures.

[12] Qi J.-N., Ma Z.J., Wang J.-Q., Liu T.-X. (2016). Post-cracking shear strength and deformability of HSS-UHPFRC beams. Struct. Concr..

[13] Pourbaba M., Joghataie A. (2018). Shear behavior of ultra-high performance concrete. Constr. Build. Mater..

[14] Mészöly T., Randl N. (2018). Shear behavior of fiber-reinforced ultra-high performance concrete beams. Eng. Struct..

[15] Kamal M.M., Safan M.A., Etman Z.A., Salama R.A. (2014). Behavior and strength of beams cast with ultra high strength concrete containing different types of fibers. HBRC J..

[16] Ngo T.T., Park J.K., Pyo S., Kim D.J. (2017). Shear resistance of ultra-high-performance fiber-reinforced concrete. Constr. Build. Mater..

[17] Voo Y.L., Poon W.K., Foster S.J. (2010). Shear strength of steel fiber-reinforced ultra high-performance concrete beams without stirrups. J. Struct. Eng..

[18] Hussein L., Amleh L. (2018). Size effect of ultra-high performance fiber reinforced concrete composite beams in shear. Struct. Concr..

[19] Ciprian T., Dan B., Victor V., Cornelia M. (2012). Ultra high performance fiber reinforced concrete I beams subjected to shear action. ACTA Technica Napocensis Civ. Eng. Archit..

[20] Baby F., Billo J., Renaud J.C., Massotte C., Marchand P., Toutlemonde F., Simon A., Lussou P., Oh B.H., Choi O.C., Chung L. (2010). Shear Resistance of Ultra High Performance Fibre-Reinforced Concrete I-Beams. Fracture Mechanics of Concrete and Concrete Structures—High Performance, Fiber Reinforced Concrete, Special Loadings and Structural Applications.

[21] Lim W.Y., Hong S.G. (2016). Shear tests for ultra-high performance fiber reinforced concrete (UHPFRC) beams with shear reinforcement. Int. J. Concr. Struct. Mater..

[22] Yoo D.-Y., Yuan T., Yang J.M., Yoon Y.S. (2017). Feasibility of replacing minimum shear reinforcement with steel fibers for sustainable high-strength concrete beams. Eng. Struct..

[23] Zagon R., Matthys S., Kiss Z. (2006). Shear behaviour of SFR-UHPC I-shaped beams. Constr. Build. Mater..

[24] Hasgul U., Yavas A., Birol T., Turker K. (2019). Steel fiber use as shear reinforcement on I-shaped UHP-FRC beams. Appl. Sci..

[25] ACI 318 (2014). Building Code Requirements for Structural Concrete (ACI 318M-14) and Commentary (318R-14).

[26] EN 1992-1-1 (2004). Eurocode 2: Design of Concrete Structures—Part 1-1: General Rules and Rules for Buildings.

[27] NZS 3101 (2006). Concrete Structures Standard—Part 1: The Design of Concrete Structures.

[28] Model Code 2010 (2013). fib Model Code for Concrete Structures 2010.

[29] BS EN 12390-3:2019 (2019). Testing Hardened Concrete. Compressive Strength of Test Specimens.

[30] ASTM C496/C496M—1 (2017). Standard Test Method for Splitting Tensile Strength of Cylindrical Concrete Specimens.

[31] ASTM C 1609/C 1609M-02 (2012). Standard Test Method for Flexural Performance of Fiber Reinforced Concrete (Using Beam with Third Point Loading).

[32] TS-500 (2000). Requirements for Design and Construction of Reinforced Concrete Structures.

[33] ASTM A370-19e1 (2019). Standard Test Methods and Definitions for Mechanical Testing of Steel Products.

[34] Yang I.H., Joh C., Kim B.S. (2010). Structural behavior of ultra high performance concrete beams subjected to bending. Eng. Struct..

[35] Yoo D.Y., Yoon Y.S. (2015). Structural performance of ultra-high-performance concrete beams with different steel fibers. Eng. Struct..

[36] Yoo D.Y., Banthia N., Yoon Y.S. (2017). Experimental and numerical study on flexural behavior of UHPFRC beams with low reinforcement ratios. Can. J. Civ. Eng..

[37] Dancygier A.N., Berkover E. (2016). Cracking localization and reduced ductility in fiber-reinforced concrete beams with low reinforcement ratios. Eng. Struct..

[38] Deluce J.R., Vecchio F.J. (2013). Cracking behavior of steel fiber-reinforced concrete members containing conventional reinforcement. ACI Struct. J..

[39] Yuguang Y., Walraven J.C., Uijl J.A. (2009). Combined effect of fibers and steel rebars in high performance concrete. Heron.

[40] Dancygier A.N., Savir Z. (2006). Flexural behavior of HSFRC with low reinforcement ratios. Eng. Struct..

[41] Fehling E., Schmidt M., Walraven J., Leutbecher T., Frönlich S. (2014). Ultra-High Performance Concrete UHPC: Fundamentals, Design, Examples, Beton-Kalender.

[42] AFGC/SETRA (2013). Recommendation: Ultra High Performance Fibre-Reinforced Concretes.

[43] JSCE (2008). Recommendations for Design and Construction of High Performance Fiber Reinforced Cement Composites with Multiple Fine Cracks (HPFRCC).

[44] Yang I.H., Joh C., Kim B.S. (2011). Flexural strength of large-scale ultra high performance concrete prestressed T-beams. Can. J. Civ. Eng..

[45] Qi J., Wang J., John Z. (2018). Flexural response of high-strength steel-ultra-high-performance fiber reinforced concrete beams based on a mesoscale constitutive model: Experiment and theory. Struct. Concr..

[46] Chen S., Zhang R., Jia L.J., Wang J.Y. (2018). Flexural behaviour of rebar-reinforced ultrahigh-performance concrete beams. Magn. Concr. Res..

[47] Khalil W., Tayfur Y.R. (2013). Flexural strength of fibrous ultra high performance reinforced concrete beams. ARPN J. Eng. Appl. Sci..

[48] Imam M., Vandewalle L., Mortelmans F. (1995). Shear-moment analysis of reinforced high strength concrete beams containing steel fibres. Can. J. Civ. Eng..

[49] Xia J., Chanb T., Mackieb K.R., Saleemc M.A., Mirmiran A. (2018). Sectional analysis for design of ultra-high performance fiber reinforced concrete beams with passive reinforcement. Eng. Struct..

[50] Lim T.Y., Paramasivam P., Lee S.L. (1987). Shear and moment capacity of reinforced steel fiber concrete beams. Magn. Concr. Res..

[51] Bae B.I., Choi H.K., Choi C.S. (2016). Flexural strength evaluation of reinforced concrete members with ultra high performance concrete. Adv. Mater. Sci. Eng..

[52] Hegger J., Bertram G., Walraven J.C., Stoelhorst D. (2008). Shear Carrying Capacity of Ultra-High Performance Concrete Beams. Tailor Made Concrete Structures.

[53] ACI 544 (2009). Design Considerations for Steel Fiber Reinforced Concrete (Reapproved 2009) (ACI 544.4R-88).

[54] Naaman E., Najm H. (1991). Bond-slip mechanisms of steel fibers in concrete. ACI Mater. J..

[55] Mansur M., Ong K., Paramasivam P. (1986). Shear strength of fibrous concrete beams without stirrups. J. Struct. Eng..

[56] Ahmad S., Bahij S., Al-Osta M.A., Adekunle S.K., Al-Dulaijan S.U. (2019). Shear behavior of ultra-high-performance concrete beams reinforced with high-strength steel bars. ACI Struct. J..

